# Impact of Hfq on the *Bacillus subtilis* Transcriptome

**DOI:** 10.1371/journal.pone.0098661

**Published:** 2014-06-16

**Authors:** Hermann Hämmerle, Fabian Amman, Branislav Večerek, Jörg Stülke, Ivo Hofacker, Udo Bläsi

**Affiliations:** 1 Department of Microbiology, Immunobiology and Genetics, Max F. Perutz Laboratories, Centre of Molecular Biology, University of Vienna, Vienna, Austria; 2 Institute for Theoretical Chemistry, University of Vienna, Vienna, Austria; 3 Department of General Microbiology, Institute of Microbiology and Genetics, Georg-August University Göttingen, Göttingen, Germany; Max-Planck-Institute for Terrestrial Microbiology, Germany

## Abstract

The RNA chaperone Hfq acts as a central player in post-transcriptional gene regulation in several Gram-negative Bacteria, whereas comparatively little is known about its role in Gram-positive Bacteria. Here, we studied the function of Hfq in *Bacillus subtilis*, and show that it confers a survival advantage. A comparative transcriptome analysis revealed mRNAs with a differential abundance that are governed by the ResD-ResE system required for aerobic and anaerobic respiration. Expression of *resD* was found to be up-regulated in the *hfq^−^* strain. Furthermore, several genes of the GerE and ComK regulons were de-regulated in the *hfq^−^* background. Surprisingly, only six out of >100 known and predicted small RNAs (sRNAs) showed altered abundance in the absence of Hfq. Moreover, Hfq positively affected the transcript abundance of genes encoding type I toxin-antitoxin systems. Taken the moderate effect on sRNA levels and mRNAs together, it seems rather unlikely that Hfq plays a central role in RNA transactions in *Bacillus subtilis*.

## Introduction

In response to environmental cues, Bacteria synthesize small regulating RNAs (sRNAs). Many sRNAs act at the post-transcriptional level through base-pairing with target mRNAs [1]. In *Escherichia coli*, the sRNA-mRNA interactions often require the RNA chaperone Hfq. Hfq belongs to the Sm and Sm-like family of proteins [2], [3]. It interacts with sRNAs and mRNAs, and assists in sRNA-mRNA annealing. Hfq-mediated riboregulation can result in post-transcriptional gene activation or silencing [3]. In addition, binding of Hfq to sRNAs can affect their stability [4]–[7]. Comparative genome analyses revealed that approximately half of the sequenced bacterial genomes contain at least one gene, encoding a Hfq orthologue [2], [8]–[9]. Throughout bacterial species, Hfq proteins comprise a conserved core but differ in length of their C-terminal extensions, with the γ- and β-Proteobacteria displaying the longest C-termini [9]. In contrast, the Hfq orthologs of Gram-positive Bacteria lack extended C-termini [2], [8]. A c-terminally truncated *E. coli* Hfq variant comprising the conserved core (aa 1–65) was unable to support translational autoregulation of the *hfq* gene as well as translational activation of the *rpoS* gene by the sRNA DsrA at low temperature [10], [11]. However, Hfq variants containing N-terminal 69 and 72 aa, respectively, were proficient in mediating riboregulation of other genes [12]. In light of these results it seems possible that some Hfq-mediated regulatory events require the extended C-terminus, whereas it seems to be dispensable for others.

The *Bacillus subtilis hfq* gene (first annotated as *ymaH*) encodes a 73 amino acid protein (Hfq*_Bs_*), forms homohexamers and was shown to bind to a RNA aptamer [13]. Hfq*_Bs_* was unable to functionally replace the *E. coli* protein *in vivo* [10]. Similarly, the presence of *Staphylococcus aureus* Hfq did not rescue Hfq-related phenotypes of a *Salmonella typhimurium hfq^−^* mutant [14]. Despite of producing Hfq*_Sa_* at levels comparable to Hfq*_St_* in wild-type *Salmonella*, the complemented strain behaved as a *hfq* null mutant in three sRNA-mediated regulatory responses. In addition, in contrast to *E. coli* Hfq, Hfq*_Bs_* did not stimulate annealing of complementary oligonucleotides, suggesting that the evolutionarily conserved core may not be sufficient to support base-pairing of two RNAs [11].

The *B. subtilis* SR1 sRNA acts by base-pairing and inhibits translation of *ahrC* mRNA, encoding a positive regulator of the arginine catabolic operons *rocABC* and *rocDEF* [15]. Although *B. subtilis* Hfq was shown to bind to both, SR1 sRNA and *ahrC* mRNA *in vitro*, it did not stabilize the SR1 sRNA *in vivo*, and was not required for duplex formation between the two RNAs. However, the expression of a translational *ahrC* reporter gene fusion was reduced in the absence of Hfq, indicating that the protein is required for efficient expression of *ahrC* mRNA *in vivo* [15], [16]. So far, no evidence for an involvement of Hfq in sRNA-mediated regulation has been obtained in *B. subtilis* [15]–[20]. Nevertheless, a deep sequencing approach of Hfq-associated RNAs revealed a subset of sRNAs, antisense RNAs, antitoxin transcripts, and many mRNA leaders that associate with Hfq [21].

In Gram-positive Bacteria, a phenotype for Hfq has only been described in *S. aureus* and *Listeria monocytogenes*. Liu *et al.* [22] showed that the deletion of *hfq* caused increased carotenoid pigmentation and a decreased toxicity of *S. aureus*, the latter of which might be attributed to the differential expression of more than 100 genes, most of which are related to stress response and virulence of *S. aureus*. However, for the RNAIII/*spa* model system it was shown that Hfq did not affect sRNA-mRNA annealing although it specifically bound to RNAIII and *spa* mRNA *in vitro* [23]. Furthermore, Hfq did not affect RNAIII or *spa* mRNA quantities *in vivo* [24]. In addition, Hfq is apparently not required for other targets of RNAIII, *sa1000* and *coa* mRNAs [24], [25]. In accordance, Hfq had no major impact on the levels of 11 sRNAs, characterized in *S. aureus* [26]. In *E. coli*, Hfq-dependent sRNA-mRNA duplexes are formed between relatively short RNA base-pairing sequences, whereas sRNA-mRNA duplexes in *S. aureus* are generally more stable [27], which could explain the dispensability for Hfq-like RNA chaperones in this organism.

In *L. monocytogenes* Hfq was shown to be important for tolerance to osmotic and ethanol stress, for long-term survival under amino acid-limiting conditions and for pathogenicity in mice [28]. Co-immunoprecipitation of Hfq-associated RNAs identified three novel sRNAs, LhrA-C [29]. Although Hfq interacted with all three sRNAs *in vitro*, only LhrA sRNA showed a reduced stability in a *L. monocytogenes hfq^−^* strain [29]. Further studies revealed that LhrA most likely acts by regulating the translation of three mRNAs, *lmo0850*, *lmo0302* and *chiA* through an anti-sense mechanism that depends on Hfq [30], [31].

*In Streptococcus pneumoniae*, close to hundred sRNAs have been identified [32]–[37]. However, most of their target genes remain unidentified. Some of the *S. pneumoniae* sRNAs are part of the regulon governed by the two-component system CiaRH [32]. Computational target predictions and verification by reporter gene assays identified six genes that are controlled by five redundant csRNAs (*c*ia-dependent *s*mall RNAs): *spr0081*, *spr0371*, *spr0159*, *spr0551*, *spr1097* and *spr2043* [37]. Thus, sRNA-mediated post-transcriptional regulation seems to occur in *S. pneumoniae*, although the family of Streptococcaceae does not contain a *hfq* homologue [9].

In this study we have performed comparative transcriptional profiling of a *B. subtilis* wild-type strain and an isogenic *hfq* deletion mutant using an RNAseq approach. In contrast to studies in Gram-negative bacteria [38], [39] the absence of Hfq had no global effects on the transcriptome. Hfq rather impacted on distinct regulons including the (i) ResD-ResE signal transduction system required for aerobic and anaerobic respiration [40], (ii) the GerE regulon encoding sigma-K-dependent late spore coat genes [41], as well as (iii) the ComK regulon, required for the regulation of competence and DNA uptake [42]. Surprisingly, only six known and predicted *B. subtilis* sRNAs showed an altered abundance. Furthermore, 3 of the down-regulated RNAs in the *hfq^−^* strain belong to the class I toxin-antitoxin (TA) systems and represent the toxin encoding mRNAs. In the absence of Hfq, the levels of their convergently transcribed antitoxin/antisense RNAs were also reduced to a similar extend as the toxin mRNAs. Thus, Hfq seems to affect both the toxin and antitoxin transcripts in a positive manner.

## Results and Discussion

### The *hfq* Gene Confers a Survival Advantage to *B. subtilis* in Stationary Phase

**Figure 1:**
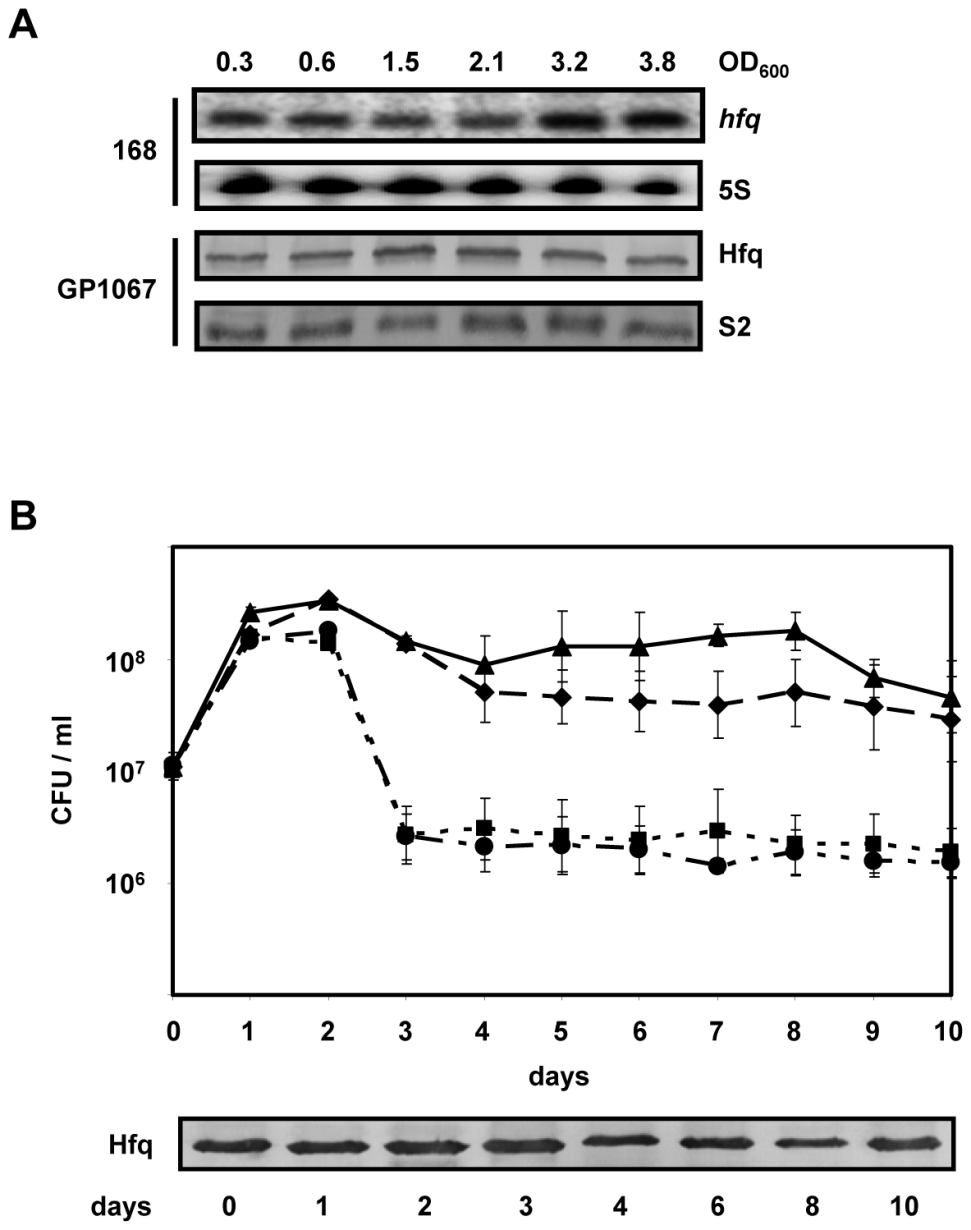
Expression of the *hfq* gene and requirement of Hfq for survival of *B. subtilis*. (A) *Bacillus subtilis* strain 168 wt and GP1067 were grown in CS-glucose medium at 37°C. The *hfq* mRNA and 5S rRNA (internal control) levels were assessed by primer extension analyses in *B. subtilis* strain 168 (upper two panels). Strain GP1067 was used to monitor Hfq and ribosomal S2 (internal control) levels by quantitative western-blotting (lower two panels). Samples for RNA extraction and for western-blot analyses were withdrawn throughout growth at the OD_600_ values indicated on top. Only the relevant sections of the autoradiographs and immunoblots are shown. (B) The *B. subtilis* strains 168 wt (triangles) and 168Δ*hfq* (squares) were co-cultivated in CS-glucose medium. Likewise, *B. subtilis* strains GP1067 (diamonds) and 168Δ*hfq* (circles) were co-cultivated in CS-glucose medium. Growth was monitored over 10 days by scoring the CFU. Immunodetection of Hfq-Flag (lower panel) in strain GP1067 was performed at the days indicated. Only the relevant section of the immunoblot is shown.

To monitor *hfq* expression throughout growth, *B. subtilis* 168 was cultivated in minimal CS-glucose medium. The *hfq* mRNA levels were determined by primer extension analysis at different times during growth (Figure 1A). In addition, Hfq synthesis was assessed in strain GP1067, encoding a *hfq::flag* fusion gene, transcription of which is driven by the authentic promoter. In minimal CS-glucose medium *hfq* was expressed in *B. subtilis* 168 during early logarithmic growth and expression only slightly increased in late stationary phase (Figure 1A). Similarly, no gross difference in the Hfq levels was observed in strain GP1067 throughout growth (Figure 1A). The latter result is at variance with a recent study [21], reporting that the synthesis of chromosomally encoded Hfq-Flag increased upon transition to stationary phase.

To test whether Hfq impacts on the viability of *B. subtilis*, strain 168Δ*hfq* was co-cultivated with the parental wild-type strain and strain GP1067, respectively, and growth was monitored in CS-glucose minimal medium at 37°C over 10 days. As shown in Figure 1B, the CFU of the 168Δ*hfq* strain started to decline at day two after inoculation. On day three, the CFU of strain 168Δ*hfq* was reduced to ∼1% when compared with the wild-type strain or with strain GP1067. As shown for other Bacteria [10], [28], [43], this experiment indicated that Hfq might play a role in survival of *B. subtilis* cells during stationary phase and prompted us to compare the transcriptomes of *B. subtilis* strains 168 and 168Δ*hfq* by RNAseq during logarithmic growth and at the onset of stationary phase.

### Transcriptional Profiling of *B. subtilis* 168Δ*hfq*

Total RNA was isolated from cultures of *B. subtilis* 168 wt and 168Δ*hfq* grown in CS-glucose minimal medium at 37°C to an OD_600_ of 0.7 and 2.0, respectively. The growth rate was indistinguishable until an OD_600_ of ∼0.7, whereas the growth rate of the *hfq^−^* strain was somewhat reduced at higher OD_600_ values (Figure S1). All genes, annotated in the NCBI database were included in the differential gene expression analysis. A p-value (adjusted for multiple testing) of 0.1 was set as threshold for significance.

**Figure 2:**
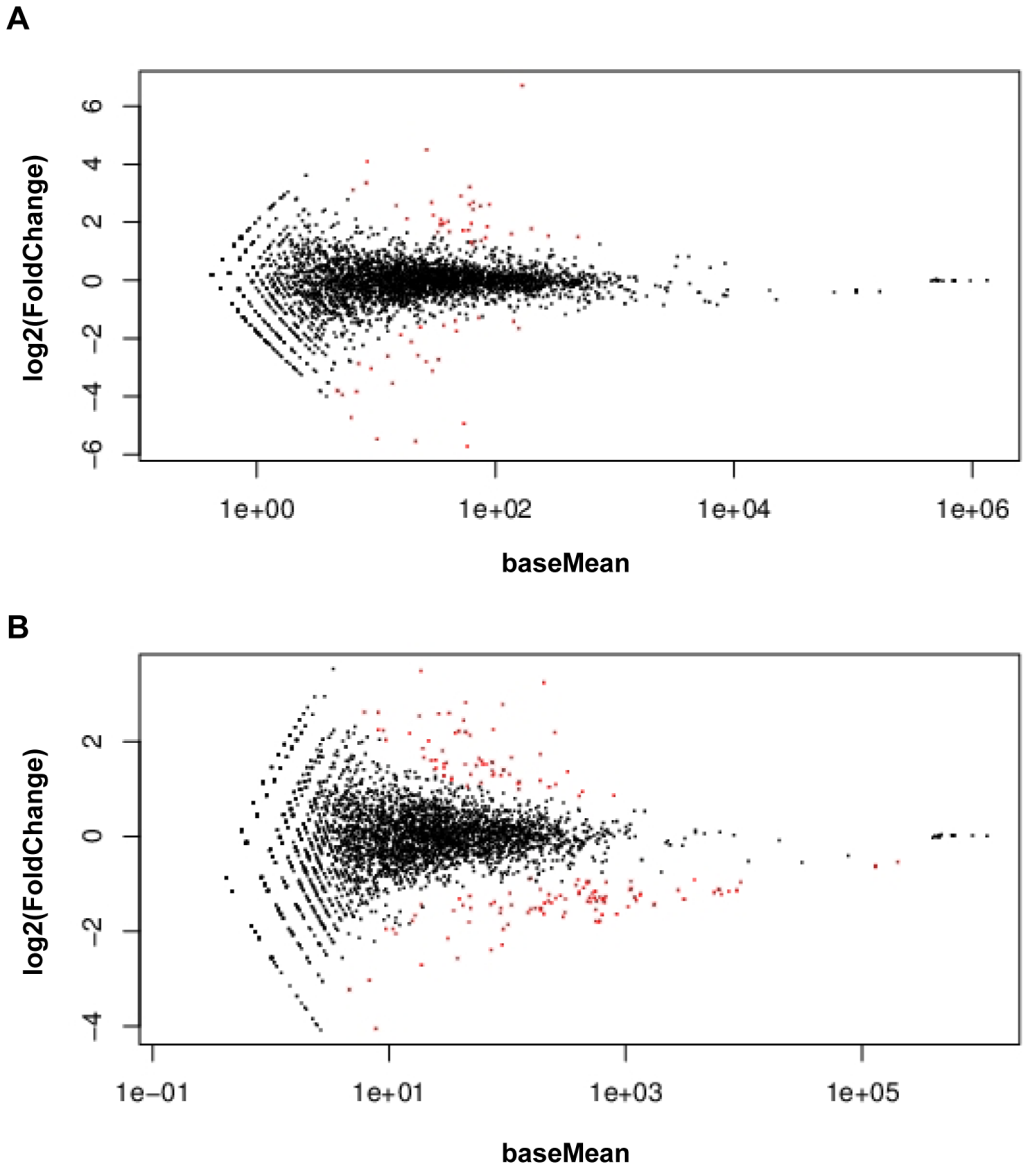
DEseq analysis of *B. subtilis* 168 wt versus 168Δ*hfq*. (A) Differential abundance of transcripts in strain 168 wt and in strain 168Δ*hfq* during logarithmic growth (OD_600_ = 0.7) (B) Differential abundance of transcripts in strain 168 wt and in strain 168Δ*hfq* in early stationary phase (OD_600_ = 2.0). Each dot represents one transcript. The log2 fold-change is plotted against the mean expression level for each transcript. Red dots represent transcripts whose abundance is significantly altered (p-value adjusted for multiple testing <0.1).

**Figure 3:**
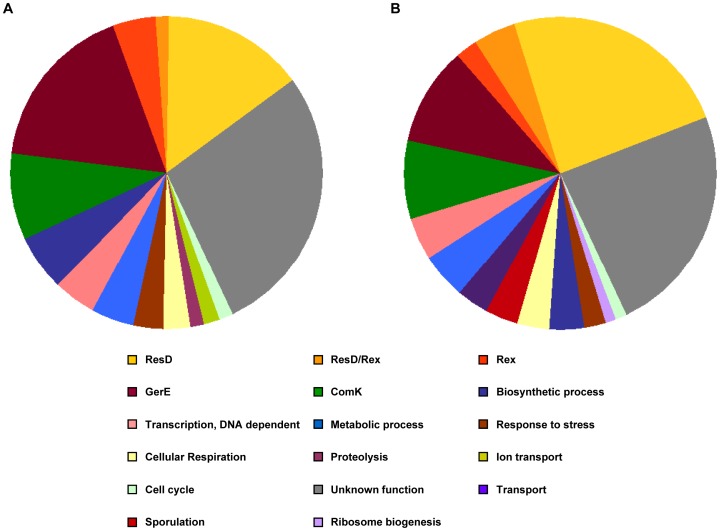
Functional classification of mRNAs with altered abundance in *B. subtilis* strain 168Δ*hfq*. (A) Gene transcripts significantly altered during logarithmic phase. (B) Gene transcripts significantly altered during early stationary phase. Genes are classified either according to their regulon affiliation (for the regulons ResD, Rex, GerE and ComK) or according to gene ontology (restricted to the terms within the domain “biological process”).

When compared with the wild-type strain 168, 68 and 88 mRNA transcripts showed a differential abundance in strain 168Δ*hfq* during logarithmic growth and in early stationary phase, respectively (Figure 2A and B). Among them, the level of 43 mRNAs was altered during both growth phases. In logarithmic growth phase 30 mRNAs were down-regulated and 38 were up-regulated (Table S1 in File S1), whereas in early stationary phase 22 were down- and 66 were up-regulated (Table S2 in File S1). A functional classification of these genes revealed that a significant number of transcripts belong to the ResD, Rex, GerE and ComK regulons (Figure 3A and B).

### Expression of Genes Involved in Anaerobic Respiration is Enhanced in the Absence of Hfq

Notably, 16.2% and 28.4% of the mRNAs with significantly altered abundance in logarithmic and early stationary phase, respectively, are governed by the ResD-ResE two component system required for aerobic and anaerobic respiration [40]. The abundance of transcripts of the *resABCDE* operon, as well as the abundance of transcripts of many ResD-regulated genes was increased in the *hfq^−^* strain (Tables S1 and S2 in File S1). ResE is a sensor kinase that, upon autophosphorylation, phosphorylates the response regulator, ResD. Subsequently, phosphorylated ResD activates transcription at target promoters. The *resD* and *resE* genes constitute an operon with the three upstream genes, *resABC* (Figure S2). The *resD/E* genes are transcribed from a *resDE*-specific promoter and the *resA* operon promoter, the latter of which is dependent on ResD/E. The ResD/E system governs anaerobic respiration by regulating the anaerobic regulator *fnr* and the *nasDEF* genes, which encode subunits of nitrite reductase [44]–[47] (Figure S2). The genes *fnr*, *nasD* and *nasE* displayed an increased transcript abundance in the *hfq^−^* strain (Tables S1 and S2 in File S1).

The genes *cydABCD*, *lctP-ldh* and *ywcJ*, which are regulated by the transcriptional regulator Rex, were as well up-regulated in the absence of Hfq (Tables S1 and S2 in File S1). The Rex repressor controls expression of the respiratory oxidase cytochrome *bd* (*cydABCD*), the NADH-linked fermentative lactate dehydrogenase (*lctP-ldh*), a type II NADH dehydrogenase (*yjlC-ndh*) and a putative nitrite transporter (*ywcJ*) [48]–[50]. The redox-sensing Rex protein indirectly responds to changes in oxygen availability and transcription of the Rex-repressed genes is activated when oxygen is limiting [48]. In the absence of oxygen, ResD-dependent genes are activated as well. As the transcript abundance of several genes of the ResD and Rex regulons was increased in the *hfq^−^* mutant, it seems likely that Hfq influences the adaptation of *B. subtilis* to anaerobic growth. This could be mediated by the ResD-ResE system as it occupies an early stage in the regulatory pathway governing anaerobic respiration.

**Figure 4:**
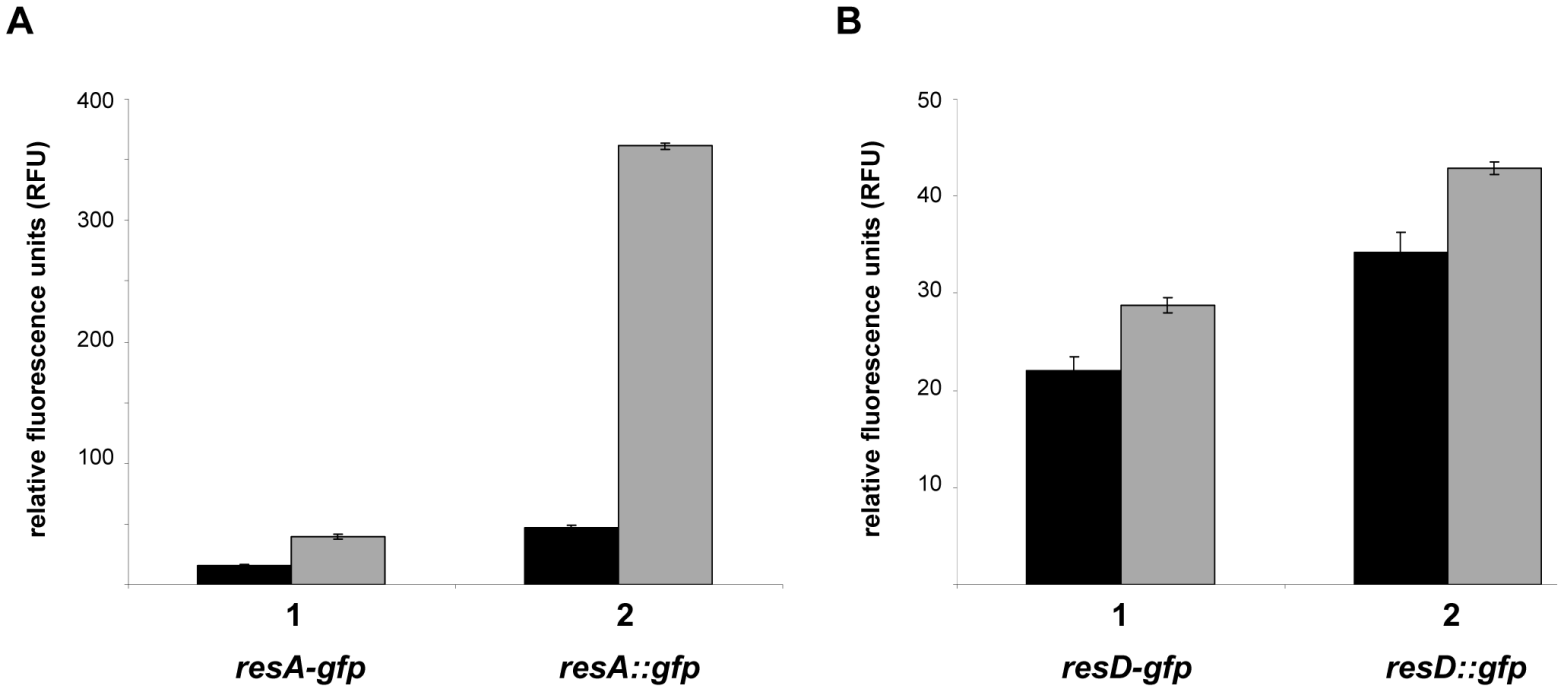
Down-regulation of *resA/D* expression in the presence of Hfq. (A) Fluorescence conferred by plasmid pADresAts (1) borne transcriptional *resA-gfp* fusion and by the plasmid pADresAtl (2) borne translational *resA::gfp* fusion, respectively, was determined in *B. subtilis* strains 168 wt (black bars) and 168Δ*hfq* (gray bars). (B) Fluorescence conferred by the plasmid pADresDts (1) borne transcriptional *resD-gfp* fusion and by the plasmid pADresDtl (2) borne translational *resD::gfp* fusion, respectively, was determined in *B. subtilis* strains 168 wt (black bars) and 168Δ*hfq* (gray bars). Error bars represent standard deviations.

To verify up-regulation of the *resD/E* transcripts in the absence of Hfq the *B. subtilis* strain 168 and the isogenic strain 168Δ*hfq* were transformed with plasmids encoding transcriptional *resA-gfp* and *resD-gfp* - and translational *resA::gfp* and *resD::gfp* fusion genes, respectively. The strains were grown in CS-glucose medium at 37°C to an OD_600_ of 0.7 when fluorescence of the cells was measured. For both genes, *resA* and *resD*, transcription was enhanced in the *hfq^−^* background when compared with the *hfq^+^* strain (Figure 4A and B), which was also mirrored by the increased translation of the *resA::gfp* and *resD::gfp* fusion genes (Figure 4A and B). As up-regulation of *resD* transcription results in elevated levels of *resA* [47], we assume that Hfq negatively impacts primarily on the transcript levels of *resD*.

**Figure 5:**
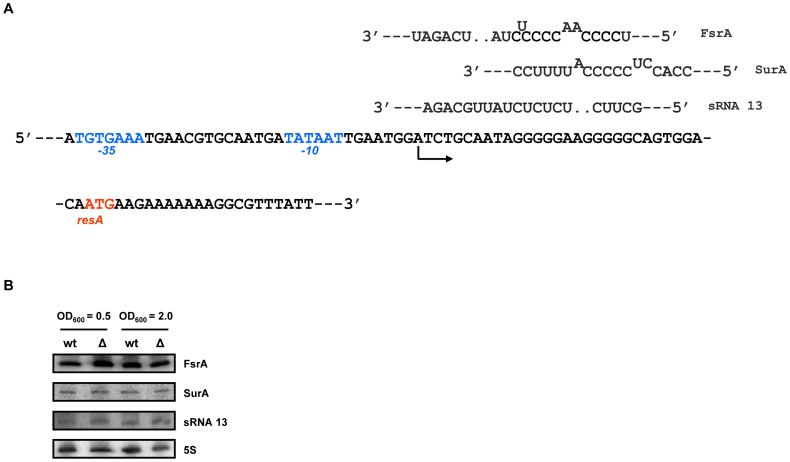
The abundance of putative sRNA regulators of *resA* is independent of Hfq. (A) Partial sequence of the 5′-untranslated region of *resA* and of the initial coding region. The −10 and −35 regions of the *resA* operon promoter are highlighted in blue. Arrow: start site and direction of transcription. The start codon (ATG) of *resA* is indicated in red. Predicted base-pairing interactions of sequences of FsrA, SurA and sRNA 13 with the rbs of *resA* mRNA. (B) The levels of FsrA, SurA, sRNA 13 and 5S rRNA (loading control) were determined by Northern-blot analyses as described in Materials and Methods. *Bacillus subtilis* strains 168 wt (wt) and 168Δ*hfq* (Δ) were grown in CS-glucose medium at 37°C. Samples for RNA extraction were withdrawn at the OD_600_ values indicated on top. Only the relevant sections of the autoradiographs are shown.

In *E. coli*, Hfq acts as a RNA chaperone, which mediates sRNA-mRNA interactions and thereby regulates the expression of target mRNAs [3]. As Hfq*_Bs_* affected the transcript abundance of the *resABCDE* operon in a negative manner (Figure 4A and B), we hypothesized that the transcripts of the *resABCDE* operon could represent targets that are post-transcriptionally regulated by Hfq and sRNAs. A bioinformatic approach was used to identify sRNAs that could possibly interact with the transcripts of the *resABCDE* operon. Verified and predicted sRNAs of *Bacillus subtilis* were used as a source for the analysis [51], [52]. Three sRNA candidates (sRNA 13 [51], FsrA [19], SurA [53], Figure 5A) with the potential to base-pair adjacent to the start codon of *resA* were identified. However, none of the sRNAs showed significantly altered levels in the RNAseq analysis, and we did not observe a difference in abundance of these sRNAs in the absence of Hfq in Northern-blot analyses (Figure 5B). The unchanged levels of FsrA observed in the *hfq^−^* mutant (Figure 5B) are at variance with recent data by Dambach *et al.* [21], who observed reduced levels of FsrA in the absence of Hfq. Transcription of FsrA is repressed by the *f*erric *u*ptake *r*egulator protein Fur [17]. Interestingly, *resA* mRNA levels were found to be decreased in a *B. subtilis fur* mutant that has elevated levels of FsrA. However, the levels of *resA* were restored to wild-type levels in *fur/fbpAB* or *fur/fbpABC* mutant strains, suggesting that the potential regulation of *resA* by FsrA is mediated by the FbpA, B and C proteins rather than by Hfq [17]. FpbA,B and C are small basic proteins, which have been proposed to function as dedicated Fur-regulated RNA chaperones [17].

### Hfq Affects the GerE and ComK regulons

17,6% and 10,2% of all mRNAs with significantly altered abundance in logarithmic and early stationary phase, respectively, belonged to the GerE regulon (Tables S1 and S2 in File S1). The regulon is controlled by the transcriptional regulator GerE and encodes a subset of sigma-K-dependent late spore coat genes [41]. The transcript levels of *gerE* and many genes governed by GerE are decreased in the *hfq^−^* strain (Tables S1 and S2 in File S1). In opposite, many genes, which were up-regulated in the presence of Hfq belonged to the ComK regulon, required for the regulation of cell competence and DNA uptake (Tables S1 and S2 in File S1) [42], [54]. Prolonged nutritional stress can result in the development of competence and sporulation [54], [55]. Towards sporulation, the individual cells can opt for the differentiated state of competence, triggered by the competence master regulator ComK [54]–[56]. In this state the cell can take up exogenous DNA from lysed cells. As Hfq impacted inversely on the ComK and GerE regulons, it could be involved in regulating steps towards sporulation.

### sRNA Abundance is Hardly Affected by Hfq

In *E. coli*, Hfq plays a central role in sRNA-mediated post-transcriptional regulation of target mRNAs, and was shown to affect the stability and half-life of several sRNAs [3]. Therefore, it was somewhat surprising that only a few *B. subtilis* sRNAs showed a significant change in abundance upon deletion of the *hfq* gene. Only six out of the >100 predicted and confirmed sRNAs of *B. subtilis* [51], [52] were found to be differentially abundant (Tables S1 and S2 in File S1). All other sRNAs did not display a significant change in abundance in the absence of Hfq as the corresponding p-values (adjusted for multiple testing) were above the threshold set for significance (>0.1). However, five of the sRNAs exhibited a decreased abundance in the absence of Hfq, whereas the level of one sRNA (sRNA 471) was increased (Tables S1 and S2 in File S1).

Similarly to this study, only 22 *B. subtilis* sRNAs were identified by Co-IP with Hfq*_Bs_* specific antibodies [21]. Correspondingly, a Co-IP approach in *Listeria monocytogenes* revealed only three sRNAs [29], whereas more than 60 sRNAs have been predicted or confirmed in *L. monocytogenes* [57]–[59]. In contrast, RNA Co-IP experiments with epitope-tagged Hfq in *Salmonella* resulted in the detection of about half of the sRNA complement in this organism [60]. In contrast to Enterobacteriaceae it seems therefore rather questionable whether Hfq plays a central role in sRNA-mediated post-transcriptional regulation in Firmicutes.

### Hfq Affects the Abundance of Toxin/Antitoxin RNAs

Among the mRNAs that exhibited a significantly reduced abundance, 3 mRNAs encode the toxin genes *bsrE*, *bsrG* and *bsrH*, which belong to type I toxin-antitoxin (TA) systems. In type I TA systems the antitoxin is an antisense RNA that is convergently transcribed with the toxin encoding mRNA [61]. Interestingly, the antisense RNAs of the *bsrE*, *bsrG* and and *txpA* TA systems were also enriched in Co-IP studies with Hfq*_Bs_* [21]. To verify the reduced levels of the three toxin mRNAs in the absence of Hfq, strain 168 and the isogenic 168Δ*hfq* strain were grown in CS-glucose medium at 37°C and samples for RNA extraction were withdrawn at an OD_600_ of 0.7 and 2.0. As verified by Northern-blot analyses, the levels of the *bsrE*, *bsrG* and *bsrH* toxin mRNAs as well as their corresponding antitoxin asRNAs as-BsrE, SR4 and as-BsrH were reduced in the absence of Hfq (Figure S3). In line with our study, the transcript levels of the type I TA systems *bsrE*/asBsrE, *bsrG*/SR4 and the RatA antitoxin were reported to be decreased in a *hfq^−^* strain [21]. In addition, our study revealed a decrease in the abundance of the transcript levels of *bsrH*/asBsrH in the absence of Hfq (Figure S3). However, Hfq neither affected the half-life of the *bsrG*/SR4 and *txpA*/RatA RNAs nor was it required for the function of the TA systems [18], [19]. Thus, it is tempting to speculate that Hfq positively affects transcription of these TA transcripts rather than their stability.

### Concluding Remarks

The comparative transcriptome analysis revealed mRNAs with a differential abundance, which are governed by the ResD-ResE system as well as several genes of the GerE and ComK regulons. Hence, it will be interesting to focus in further studies on the role of Hfq in adaptation to anaerobic growth and whether it affects competence and sporulation processes. The mRNAs/asRNAs of several type I TA systems were reduced in the absence of Hfq. As these TA systems have been hypothesized to confer an advantage to cells during stress conditions, it seems further interesting to ask whether there is a link between the reduced viability of the *hfq−* strain (Fig. 1B) and the reduced expression of the type I TA systems.

The comparison of the transcripts displaying an altered abundance in the absence of Hfq (Tables S1 and S2 in File S1) with mRNAs that associated with Hfq*_Bs_* [21] revealed significant differences. Approximately 150 Hfq-bound mRNAs fragments were detected in the Co-IP screen. However, only five mRNAs were identified in both approaches. Notably, of the five genes identified in both studies, two (*ctaD* and *sboA*) are regulated by the ResD-ResE signal transduction system, while the other three genes (*yxiE*, *yebD* and *ydbL*) are of unknown function. It is conceivable that the altered levels of many transcripts in the absence of Hfq result from a perturbation of the expression levels of a limited number of regulatory genes, and thus from indirect effects. In agreement with this is the observation that the abundance of transcripts of distinct regulons was affected (Fig. 3A and B).

## Materials and Methods

### Bacterial Strains, Growth Conditions and Transformation

The strains used in this study are listed in Table S3 in File S1. All *B. subtilis* strains used in this study are derivatives of the wild-type strain 168. *Escherichia coli* TOP10 (Invitrogen) was used for the cloning experiments. *B. subtilis* was grown in Luria Bertani (LB) medium or C-minimal medium with succinate [62] in the presence of 0.5% glucose (CS-glucose medium) supplemented with chloramphenicol (5 µg/ml), spectinomycin (150 µg/ml) or phleomycin (2.5 µg/ml), where appropriate.

The *B. subtilis* strain 168Δ*hfq* was constructed by transformation of chromosomal DNA of *B. subtilis* strain DB104Δ*hfq*::*phle* [15], which was kindly provided by Dr. Sabine Brantl. The deletion was accomplished by a double crossover of the antibiotic marker. *B. subtilis* strain GP1067 was constructed by transformation of the integrative plasmid pGP1331*hfq*short into *B. subtilis* strain 168. Strain GP1067 encodes a chromosomal *hfq::flag* fusion gene (C-terminal Flag-tag), which is controlled by the authentic promoter. The chromosomal insertions were verified by means of PCR and subsequent DNA sequencing.

*B. subtilis* was transformed with DNA according to a two-step protocol as described [63]. Transformants were selected on agar containing chloramphenicol (5 µg/ml), spectinomycin (150 µg/ml) or phleomycin (2.5 µg/ml).

### Construction of Plasmids

The oligonucleotides used for cloning are listed in Table S4 in File S1. The plasmids used in this study are based on the Gram-positive - *E. coli* shuttle vector pAD123 [64], provided by the *Bacillus Genetic Stock Center* (*BGSC*). To remove the Shine and Dalgarno sequence of the *gfp*mut3a gene of plasmid pAD123, a DNA fragment was generated by means of PCR using the forward primer V67, which contained an *Xba*I site, and the reverse primer W67, which contained a *Hind*III site. The PCR product was cleaved with *Xba*I and *Hind*III, and then ligated into the corresponding sites of plasmid pAD123 resulting in plasmid pADΔRBS.

The plasmids pADresAts and pADresDts, bearing transcriptional *resA-gfp* and *resD-gfp* fusions, are derivatives of plasmid pAD123. Construction of plasmid pADresAts: the forward primer F81, which contained an *Eco*RI site, and the reverse primer I88, which contained a *Xba*I site, were used to amplify the region spanning nucleotides −101 to +4 with regard to the transcriptional start of *resA*. The resulting PCR fragment was cleaved with *Eco*RI and *Xba*I, and then cloned into the corresponding sites of plasmid pAD123. Construction of plasmid pADresDts: the forward primer J88, which contained an *Eco*RI site, and the reverse primer L88, which contained a *Xba*I site, were used to amplify the region spanning nucleotides −100 to +4 with regard to the transcriptional start of *resD*. The resulting PCR fragment was cleaved with *Eco*RI and *Xba*I, and then cloned into the corresponding sites of plasmid pAD123.

The plasmids pADresAtl and pADresDtl, bearing translational *resA::gfp* and *resD::gfp* fusions, are derivatives of plasmid pADΔRBS. Construction of plasmid pADresAtl: the forward primer F81, which contained an *Eco*RI site, and the reverse primer G81, which contained a *Xba*I site, were used to amplify the region spanning nucleotides −131 to +18 with regard to the A(+1) of the start codon of *resA*. The resulting PCR fragment was cleaved with *Eco*RI and *Xba*I and then cloned into the corresponding sites of plasmid pADΔRBS. Construction of plasmid pADresDtl: the forward primer J88, containing an *Eco*RI site, and the reverse primer K88, containing a *Xba*I site, were used to amplify the region spanning nucleotides −124 to +18 with regard to the A(+1) of the start codon of *resD*. The resulting PCR fragment was cleaved with *Eco*RI and *Xba*I and then cloned into the corresponding sites of plasmid pADΔRBS.

The plasmid pGP1331*hfq*short used for construction of *B. subtilis* strain GP1067 is based on the integrative plasmid pGP1331 [65]. Plasmid pGP1331 was used to abut the 3′-end of *hfq* with the sequence encoding a triple Flag-tag. The *hfq::flag_(3)_* fusion gene is under transcription control of the authentic promoter. Construction of plasmid pGP1331*hfq*short: the forward primer ML152, containing a *Bam*HI site, and the reverse primer ML151, containing a *Sal*I site, were used to amplify the *hfq* gene. The resulting PCR fragment was cleaved with *Bam*HI and *Sal*I, and then cloned into the corresponding sites of plasmid pGP1331.

### Growth Competition Experiment

*B. subtilis* strain 168 wt and strain 168Δ*hfq* and the strains GP1067, encoding the *hfq::flag* fusion gene, and 168Δ*hfq*, respectively, were grown concomitantly in CS-glucose medium at 37°C. The strains were inoculated at an initial CFU/ml of 1×10^7^, and growth was followed over 10 days by scoring the colony forming units (CFU). Each day serial dilutions were plated on LB agar plates containing appropriate antibiotics for selection. To verify synthesis of Hfq in strain GP1067 during the course of the experiment, samples for western-blot analysis were withdrawn at the times indicated in Fig. 1B.

### Western-blot Analyses

The cellular levels of Hfq and S2 ribosomal protein were determined by quantitative immunoblotting. *B. subtilis* strain GP1067, encoding the Hfq-Flag-tag fusion protein, was grown in CS-glucose medium at 37°C. Samples were withdrawn at various times (indicated in Fig. 1A and B) and the cells were pelleted by centrifugation. The cell pellets were resuspended in 50 µl lysis buffer (1x TE buffer, 20 mM EDTA, 1 mg/ml lysozyme) per ml cell culture and incubated at 37°C for 20 min. At an OD_600_ of 1.0, 2 µl of 20% SDS were added, and the samples were heated at 95°C for 5 min. Then, 1 volume of 2 x protein sample buffer was added and the suspension was heated for additional 5 min at 95°C. Equal amounts of total protein were separated on 12% SDS–polyacrylamide gels, and then blotted to a nitrocellulose membrane (GE Healthcare). The blots were blocked with 5% dry milk in TBS buffer, and then probed with anti-Flag (Roth) or anti-S2 (lab stock) antibodies. The antibody-antigen complexes were visualized with goat anti-rabbit immunoglobulin alkaline-phosphatase conjugated antibody (Sigma) using NBT (Nitroblue-tetrazolium-chloride; Sigma) and BCIP (5-Bromo-4-chloro-3-indolyl phosphate toluidine salt; Sigma) in alkaline phosphatase-buffer (10 mM NaCl, 5 mM MgCl_2_, 100 mM Tris/HCl, pH 9.5) as a chromogenic substrate.

### Comparative Transcriptome Analysis

The *B. subtilis* strain 168 and strain 168Δ*hfq* were grown in CS-glucose medium at 37°C. Total RNA from logarithmically growing cultures (OD_600_ = 0.7) and cultures grown to early stationary phase (OD_600_ = 2.0) were prepared as follows. Total RNA was isolated using the Trizol method (Ambion). The samples were treated with DNase I (DNase I, RNase-free, Roche Applied Science). Next, a control PCR was performed using the *aprE*-specific oligonucleotides M70 and N70 (Table S4 in File S1) to confirm complete degradation of chromosomal DNA. The RNAseq analysis included 2 biological and 2 technical replicates. The RNA was fragmented to an average length of 200–300 nt by incubation for 2 minutes at 94°C in 40 mM Tris-acetate pH 8.2, 100 mM potassium-acetate and 30 mM magnesium-acetate [66]. The samples were first cooled on ice and then purified on a Sephadex G50 column (GE Healthcare). The cDNA synthesis was carried out using the SuperScript Double-Stranded cDNA Synthesis Kit (Invitrogen) following the manufacturer's instructions. The cDNA was purified using phenol/CHCl_3_. The cDNA of the different samples was further processed and subjected to next generation RNA sequencing (NGS; Illumina platform GAIIx) at the Vienna Biocenter Campus Science Support Facility (http://www.csf.ac.at). The protocol for cDNA samples processing and RNA sequencing is available at http://www.csf.ac.at/facilities/ngs/protocols.

### Bioinformatic Analyses

The obtained sequencing reads were mapped on the genome of *Bacillus subtilis* 168 (NC_000964.3) using Segemehl (version 0.9.3) with default parameters [67]. For each annotated mRNA from the NCBI database the number of mapped reads, for each growth phase and separately for each of the replicates, were determined by counting all reads with an overlap of at least 1 nt. To identify transcripts with different abundance in *B. subtilis* 168 and 168Δ*hfq* during logarithmic growth or at the onset of stationary phase, a differential gene expression analysis was performed, using the tool DEseq (version 1.5), which is part of the bioconductor packages [68]. A p-value (adjusted for multiple testing) below 0.1 was set as threshold for significance.

### Target Prediction for sRNAs

The *B. subtilis* sRNAs identified and predicted in recent studies [50]–[51] were used as a source for target prediction. The RNA local folding opening energy (a measure for the accessibility of the sequence and the binding sites) was calculated for pair probabilities using the program RNAplfold [69] of the ViennaRNA package with the options -W 200 -L 150 [70]. The putative binding sites between sRNA and mRNA targets were calculated using RNAplex software with the options -A -a -l 30 [71].

### Primer Extension Analyses

The levels of *hfq* mRNA and 5S ribosomal RNA were determined by primer extension. *B. subtilis* wt strain 168 was grown in CS-glucose medium at 37°C. Aliquots for RNA purification were withdrawn at the OD_600_ values indicated in Fig. 1A. The total RNA was purified with the Trizol method (Ambion). The primer extension analysis was performed using 15 µg of total RNA. After annealing to the *hfq*-specific [^32^P] 5′-end-labelled (Amersham Pharmacia Biotech) oligonucleotide Q65 (Table S4 in File S1) and addition of AMV-Reverse Transcriptase (Promega), cDNA synthesis was carried out for 15 min at 45°C. The oligonucleotide Q65 is complementary to nt +23 to +45 with regard to the A (+1) of the AUG start codon of *hfq* mRNA. Primer extension yielded a product of 80 nt in length. As a loading control, the 5S rRNA levels were determined in the corresponding RNA samples by primer extension using the 5S rRNA-specific [^32^P] 5′-end-labeled oligonucleotide B64 (Table S4 in File S1). The oligonucleotide B64 is complementary to nt +58 to +79 with regard to the transcription start site (+1) of 5S rRNA. Primer extension yielded a product of 79 nt in length. The extension products were resolved on a 8% polyacrylamide-8 M urea gel and were then visualized with a PhosphorImager (Molecular Dynamics).

### Northern-blot Analyses

The steady-state levels of the sRNAs 13, FsrA and SurA were determined by Northern-blot analyses. *B. subtilis* strain 168 and strain 168Δ*hfq* were grown in CS-glucose medium at 37°C. Aliquots for RNA purification were withdrawn at the OD_600_ values indicated in Fig. 5B. RNA extraction was performed using the Trizol method (Ambion). 15 µg of total RNA were denatured for 5 min at 85°C in RNA loading dye, separated on 8% polyacrylamide-8 M urea gels, and then transferred to a nylon membrane (Amersham Hybond-N) by electroblotting. The RNA was cross-linked to the membrane by exposure to UV light. Target-specific [γ-^32^P]-5′-end-labeled (Amersham Pharmacia Biotech) oligonucleotides were used as indicated in Table S4 in File S1. sRNA 13 was detected using an internally radiolabelled probe synthesized by means of PCR in the presence of [α-^32^P]-dATP (Amersham Pharmacia Biotech) using primers M80/N80 (Table S4 in File S1). The hybridization signals were visualized using a PhosphorImager (Molecular Dynamics). The experiments were performed in duplicate.

### Gfp-assays

*B. subtilis* strains 168 and 168Δ*hfq* bearing either plasmid pADresAts, pADresDts, pADresAtl or pADresDtl were grown in CS-glucose medium at 37°C to an OD_600_ of 0.7, at which time 1 ml aliquots were withdrawn in duplicate. The samples were pelleted by centrifugation and the pellets were dissolved in 2 ml 1x TE buffer to measure the fluorescence with a spectro-fluorometer (Jasco FP-6300) at wavelengths Ex-501 nm and Em-511 nm. The experiment was performed in triplicate.

## Supporting Information

Figure S1 **Growth and sampling of the** ***B. subtilis*** **strains for RNA-seq analysis.** *Bacillus subtilis* strains 168 wt (triangles) and 168Δ*hfq* (squares) were grown in CS-glucose medium at 37°C. Samples for RNA extraction were withdrawn at an OD_600_ of 0.7 and 2.0 as indicated by the arrows.
(TIF)

Figure S2 **Schematic representation of the** ***resABCDE*** **operon and regulation by ResD of anaerobic respiration in** ***B. subtilis*** **.** Upon autophosphorylation ResE transfers a phosphate to ResD. Subsequently, phosphorylated ResD activates transcription at target promoters. Activated genes include besides others *fnr*, encoding the anaerobic transcriptional regulator, *nasDEF*, which constitute an operon encoding the subunits of nitrite reductase, *nasBC*, nitrate reductase, *hmp*, flavohemoglobin and *narK*, nitrite extrusion protein. Shaded boxes: genes involved in anaerobic regulation; Arrows: regulatory flows; Arrows above boxes denote start sites and the direction of transcription; Red stars indicate an increased transcript abundance in the *B. subtilis* 168Δ*hfq* mutant (see Tables S1 and S2 in File S1). Adapted from Nakano and Zuber (1998) *Ann. Rev. Microbiol.* 52: 165–190.
(TIF)

Figure S3 **The steady state levels of type I TA system RNAs are reduced in the absence of Hfq.** *Bacillus subtilis* strains 168 wt (wt) and 168Δ*hfq* (Δ) were grown in CS-glucose medium at 37°C. Samples for RNA extraction were withdrawn at the OD_600_ values indicated on top. RNA extraction was performed using the Trizol method (Ambion). 15 µg of total RNA were denatured for 5 min at 85°C in RNA loading dye, separated on 8% polyacrylamide-8 M urea gels, and then transferred to a nylon membrane (Amersham Hybond-N) by electroblotting. The RNA was cross-linked to the membrane by exposure to UV light. The membrane was hybridized with target-specific [γ-^32^P]-5′-end-labeled (Amersham Pharmacia Biotech) oligonucleotides as indicated in Table S4 in File S1. The hybridization signals were visualized using a PhosphorImager (Molecular Dynamics). Only the relevant sections of the autoradiographs are shown.
(TIF)

File S1 **Supplemental Tables.** Transcripts displaying altered abundance in the absence of Hfq during logarithmic growth (Table S1) and in early stationary phase (Table S2). Bacillus subtilis strains used in this study (Table S3). Oligonucleotides used in this study (Table S4).
(DOC)
