# Supplementary figures and images for: Impact of Hfq on the Bacillus subtilis Transcriptome
Source: PLoS One. 2014 Jun 16;9(6):e98661. doi: 10.1371/journal.pone.0098661 (PMC4059632; doi:10.1371/journal.pone.0098661)

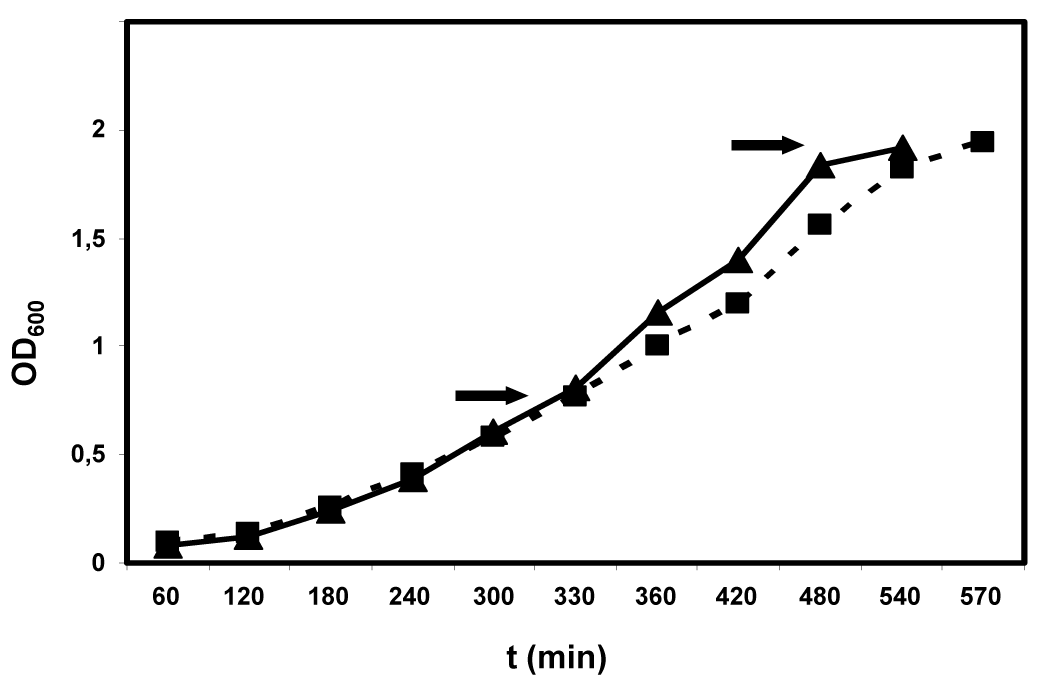

Supplement: Figure S1 — Growth and sampling of the B. subtilis strains for RNA-seq analysis. Bacillus subtilis strains 168 wt (triangles) and 168Δhfq (squares) were grown in CS-glucose medium at 37°C. Samples for RNA extraction were withdrawn at an OD600 of 0.7 and 2.0 as indicated by the arrows. (TIF) [file pone.0098661.s001.tif]

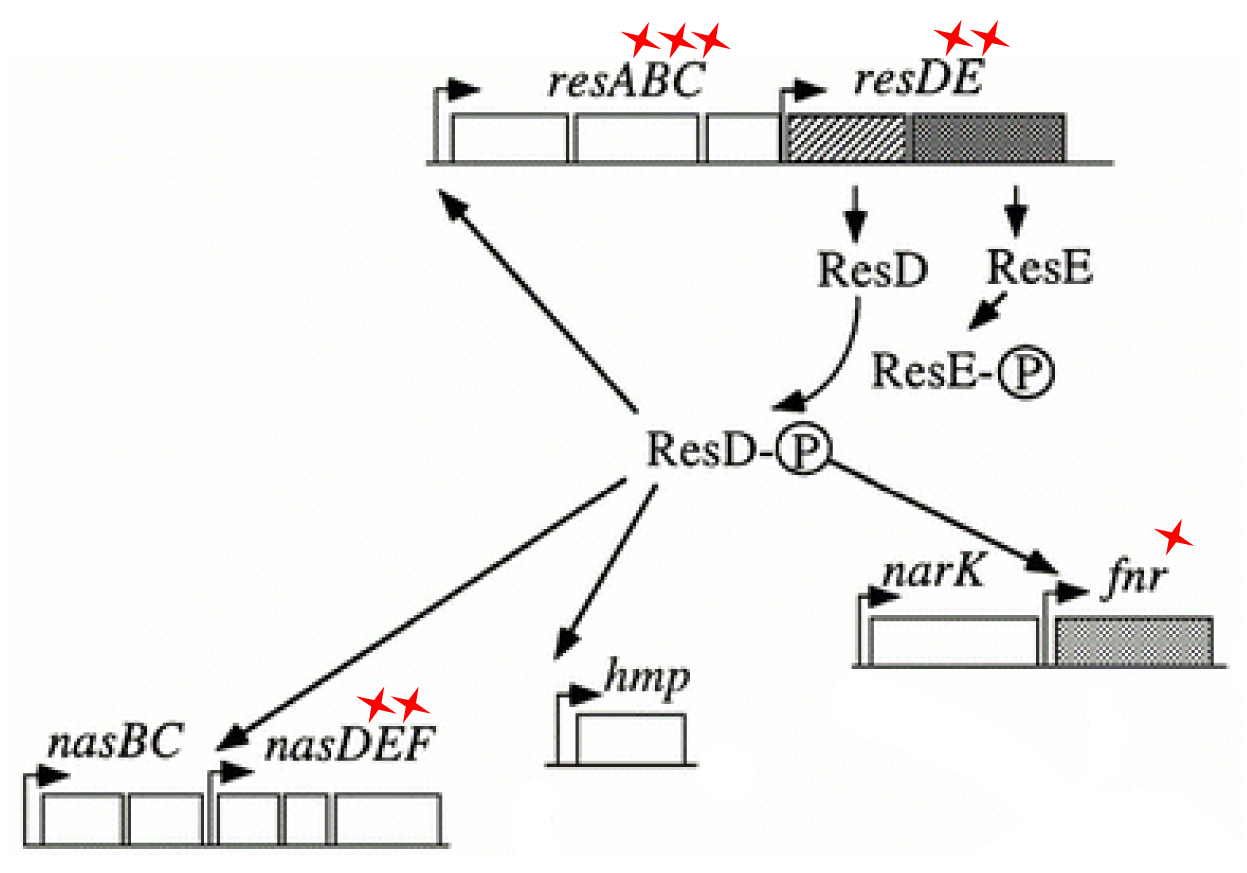

Supplement: Figure S2 — Schematic representation of the resABCDE operon and regulation by ResD of anaerobic respiration in B. subtilis . Upon autophosphorylation ResE transfers a phosphate to ResD. Subsequently, phosphorylated ResD activates transcription at target promoters. Activated genes include besides others fnr, encoding the anaerobic transcriptional regulator, nasDEF, which constitute an operon encoding the subunits of nitrite reductase, nasBC, nitrate reductase, hmp, flavohemoglobin and narK, nitrite extrusion protein. Shaded boxes: genes involved in anaerobic regulation; Arrows: regulatory flows; Arrows above boxes denote start sites and the direction of transcription; Red stars indicate an increased transcript abundance in the B. subtilis 168Δhfq mutant (see Tables S1 and S2 in File S1). Adapted from Nakano and Zuber (1998) Ann. Rev. Microbiol. 52: 165–190. (TIF) [file pone.0098661.s002.tif]

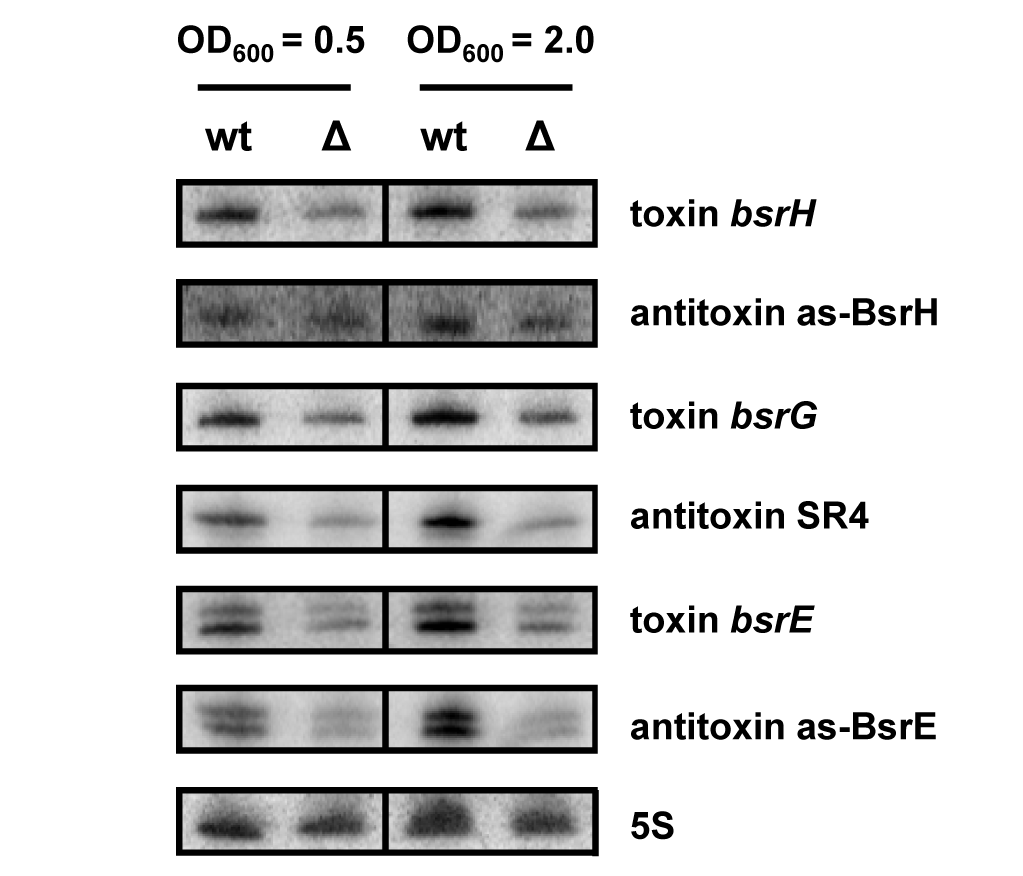

Supplement: Figure S3 — The steady state levels of type I TA system RNAs are reduced in the absence of Hfq. Bacillus subtilis strains 168 wt (wt) and 168Δhfq (Δ) were grown in CS-glucose medium at 37°C. Samples for RNA extraction were withdrawn at the OD600 values indicated on top. RNA extraction was performed using the Trizol method (Ambion). 15 µg of total RNA were denatured for 5 min at 85°C in RNA loading dye, separated on 8% polyacrylamide-8 M urea gels, and then transferred to a nylon membrane (Amersham Hybond-N) by electroblotting. The RNA was cross-linked to the membrane by exposure to UV light. The membrane was hybridized with target-specific [γ-32P]-5′-end-labeled (Amersham Pharmacia Biotech) oligonucleotides as indicated in Table S4 in File S1. The hybridization signals were visualized using a PhosphorImager (Molecular Dynamics). Only the relevant sections of the autoradiographs are shown. (TIF) [file pone.0098661.s003.tif]
