# Supplementary material for: Impact of Hfq on the Bacillus subtilis Transcriptome
Source: PLoS One. 2014 Jun 16;9(6):e98661. doi: 10.1371/journal.pone.0098661 (PMC4059632; doi:10.1371/journal.pone.0098661)
Supplement: File S1 — Supplemental Tables. Transcripts displaying altered abundance in the absence of Hfq during logarithmic growth (Table S1) and in early stationary phase (Table S2). Bacillus subtilis strains used in this study (Table S3). Oligonucleotides used in this study (Table S4). (DOC) [file pone.0098661.s004.doc]

**Supplementary Tables**

**Table S1.** **Transcripts displaying altered abundance in the absence of Hfq during logarithmic growth.** p-value adjusted for multiple testing < 0.1. Transcripts are assigned to different regulons or according to their gene ontology.

| **Regulon/GO-term** | **gene** | **log2 fold change (*hfq^-^* *vs.* wt)** | **p-value (adjusted)** |
| --- | --- | --- | --- |
|  |  |  |  |
| **ResD-regulon** |  |  |  |
|  | *nasD* | 3,21 | 3,98E-10 |
|  | *ctaA* | 2,61 | 1,12E-06 |
|  | *ctaB1* | 2,43 | 4,90E-06 |
|  | *fnr* | Inf | 0,00091 |
|  | *sboX* | 1,95 | 0,0011 |
|  | *albA* | 1,77 | 0,0029 |
|  | *sboA* | 1,60 | 0,0064 |
|  | *resC* | 1,66 | 0,014 |
|  | *resA* | 1,66 | 0,052 |
|  | *resB* | 1,36 | 0,06 |
| **ResD/Rex-regulon** |  |  |  |
|  | *cydA* | 4,50 | 1,51E-08 |
| **Rex-regulon** |  |  |  |
|  | *ldh* | 6,71 | 8,91E-27 |
|  | *ywcJ* | Inf | 0,0019 |
|  | *lctP* | 2,09 | 0,0038 |
| **GerE-regulon** |  |  |  |
|  | *cotG* | -5,72 | 1,80E-20 |
|  | *cotX* | -4,95 | 4,53E-18 |
|  | *sscA* | -5,55 | 1,69E-10 |
|  | *cotV* | -3,12 | 1,98E-07 |
|  | *cgeB* | -2,80 | 6,13E-06 |
|  | *yurS* | -5,47 | 1,01E-05 |
|  | *cgeA* | -3,54 | 2,86E-05 |
|  | *gerE* | -2,59 | 0,00012 |
|  | *cotD* | -1,66 | 0,00064 |
|  | *cotW* | -Inf | 0,00075 |
|  | *cotB* | -3,83 | 0,01 |
|  | *sspG* | -3,79 | 0,059 |
| **ComK-regulon** |  |  |  |
|  | *comGA* | 2,55 | 2,23E-07 |
|  | *comGB* | 2,23 | 0,00064 |
|  | *comFA* | 2,02 | 0,00083 |
|  | *comGC* | 2,56 | 0,0019 |
|  | *ssbB* | 1,47 | 0,006 |
|  | *comGD* | 3,11 | 0,093 |
| **Biosynthetic process** |  |  |  |
|  | *hemH* | 1,71 | 0,0038 |
|  | *hemE* | 1,93 | 0,0051 |
|  | *spsB* | -Inf | 0,064 |
|  | *hisA* | -1,29 | 0,092 |
|  |  |  |  |
|  |  |  |  |
|  |  |  |  |
|  |  |  |  |
|  |  |  |  |
| **Regulon/GO-term** | **gene** | **log2 fold change (*hfq^-^* *vs.* wt)** | **p-value (adjusted)** |
|  |  |  |  |
| **Transcription, DNA dependent** |  |  |  |
|  | *ycnK* | 1,94 | 0,0022 |
|  | *spoVIF* | -3,95 | 0,024 |
|  | *ytlI* | -3,04 | 0,057 |
| **Metabolic process** |  |  |  |
|  | *xis* | -1,76 | 0,0065 |
|  | *argF* | -1,42 | 0,023 |
|  | *csn* | 1,25 | 0,088 |
| **Response to stress** |  |  |  |
|  | *yxiE* | 1,90 | 0,0038 |
|  | *groS* | -1,40 | 0,036 |
| **Cellular respiration** |  |  |  |
|  | *qoxA* | 1,52 | 0,02 |
|  | *qoxB* | 1,49 | 0,066 |
| **Cell cycle** |  |  |  |
|  | *ydcQ* | -2,73 | 8,16E-06 |
| **Ion transport** |  |  |  |
|  | *ycnJ* | 1,85 | 0,00099 |
| **Proteolysis** |  |  |  |
|  | *aprX* | -Inf | 0,018 |
| **Unknown function** |  |  |  |
|  | *yozB* | 2,60 | 1,64E-07 |
|  | *ydbL* | 2,90 | 1,92E-07 |
|  | *yerA* | 2,69 | 5,33E-07 |
|  | *yfmQ* | 2,67 | 2,28E-05 |
|  | *yjcZ* | -Inf | 0,0015 |
|  | *yjdB* | 1,96 | 0,0019 |
|  | *yoqM* | 4,10 | 0,002 |
|  | *yqxI* | 1,73 | 0,0038 |
|  | *ydzL* | -2,11 | 0,0071 |
|  | *ydzA* | 3,36 | 0,012 |
|  | *ydcO* | -2,61 | 0,012 |
|  | *yhcO* | -Inf | 0,013 |
|  | *ytkA* | 2,12 | 0,018 |
|  | *usd* | -4,73 | 0,022 |
|  | *yczN* | -2,87 | 0,027 |
|  | *yoyG* | -1,63 | 0,042 |
|  | *ykzV* | -3,82 | 0,059 |
|  | *ydcP* | -1,89 | 0,068 |
|  | *ydbN* | 1,31 | 0,09 |
| **sRNAs** |  |  |  |
|  | sRNA 1670 | -1.58 | 0.046 |
| **Type I toxin RNAs** |  |  |  |
|  | *bsrH* | -1.38 | 0.022 |
|  | *bsrE* | -1.32 | 0.03 |
|  | *bsrG* | -1.27 | 0.07 |

**Table S2.** **Transcripts displaying altered abundance in the absence of Hfq in early stationary phase.** p-value adjusted for multiple testing < 0.1. Transcripts are assigned to different regulons or according to their gene ontology.

| **Regulon/GO-term** | **gene** | **log2 fold change (*hfq^-^* *vs.* wt)** | **p-value (adjusted)** |
| --- | --- | --- | --- |
|  |  |  |  |
| **ResD-regulon** |  |  |  |
|  | *sboA* | 3,24 | 8,77E-16 |
|  | *sboX* | 2,78 | 1,54E-09 |
|  | *albA* | 2,19 | 2,60E-09 |
|  | *albF* | 2,82 | 6,89E-08 |
|  | *ctaA* | 2,25 | 2,24E-06 |
|  | *albE* | 2,14 | 1,33E-05 |
|  | *albB* | 2,19 | 1,53E-05 |
|  | *albC* | 2,22 | 2,18E-05 |
|  | *albD* | 2,19 | 4,09E-05 |
|  | *ctaB1* | 2,21 | 8,45E-05 |
|  | *nasD* | 1,52 | 0,00066 |
|  | *albG* | 2,18 | 0,0022 |
|  | *ctaC* | 1,53 | 0,0027 |
|  | *ctaD* | 1,31 | 0,004 |
|  | *resB* | 1,64 | 0,004 |
|  | *ctaE* | 1,31 | 0,019 |
|  | *resD* | 1,53 | 0,019 |
|  | *ctaB2* | 1,13 | 0,021 |
|  | *resC* | 1,45 | 0,025 |
|  | *fnr* | 2,62 | 0,042 |
|  | *nasE* | 2,24 | 0,058 |
| **ResD/Rex-regulon** |  |  |  |
|  | *cydD* | 3,49 | 9,93E-07 |
|  | *cydC* | 2,60 | 9,93E-07 |
|  | *cydB* | 2,59 | 5,96E-06 |
|  | *cydA* | 1,55 | 0,00018 |
| **Rex-regulon** |  |  |  |
|  | *lctP* | 1,37 | 2,25E-05 |
|  | *ldh* | 0,86 | 0,002 |
| **GerE-regulon** |  |  |  |
|  | *cotG* | -2,29 | 1,24E-06 |
|  | *cgeB* | -2,57 | 1,69E-05 |
|  | *cotX* | -1,86 | 0,00034 |
|  | *gerE* | -2,16 | 0,00066 |
|  | *cotZ* | -2,71 | 0,0015 |
|  | *cgeA* | -1,78 | 0,011 |
|  | *cotD* | -1,22 | 0,013 |
|  | *sscA* | -1,57 | 0,015 |
|  | *cotV* | -1,51 | 0,029 |
| **ComK-regulon** |  |  |  |
|  | *comGA* | 1,74 | 5,33E-05 |
|  | *comEA* | 2,01 | 0,0021 |
|  | *comFA* | 1,42 | 0,0061 |
|  | *comGB* | 1,39 | 0,0066 |
|  | *ssbB* | 1,12 | 0,039 |
|  | *comGC* | 1,67 | 0,044 |
|  | *comEC* | 1,61 | 0,049 |
|  |  |  |  |
| **Regulon/GO-term** | **gene** | **log2 fold change (*hfq^-^* *vs.* wt)** | **p-value (adjusted)** |
|  |  |  |  |
| **Biosynthetic process** |  |  |  |
|  | *hemY* | 1,81 | 0,0022 |
|  | *hemA* | 1,00 | 0,046 |
|  | *hemH* | 1,07 | 0,082 |
| **Transcription, DNA dependent** |  |  |  |
|  | *sdpR* | 1,10 | 0,011 |
|  | *ycnK* | 1,56 | 0,012 |
|  | *kipR* | -3,23 | 0,039 |
|  | *ybbH* | -1,66 | 0,047 |
| **Metabolic process** |  |  |  |
|  | *csn* | 1,57 | 0,002 |
|  | *yocH* | 1,40 | 0,0051 |
|  | *ganA* | -3,23 | 0,039 |
|  | *cccB* | 1,29 | 0,093 |
| **Response to stress** |  |  |  |
|  | *yxiE* | 1,53 | 0,0021 |
|  | *mtnK* | -0,88 | 0,067 |
| **Cellular respiration** |  |  |  |
|  | *qoxC* | 1,18 | 0,0066 |
|  | *qoxA* | 1,05 | 0,025 |
|  | *qoxB* | 0,95 | 0,027 |
| **Cell cycle** |  |  |  |
|  | *ydcQ* | -1,44 | 0,087 |
| **Transport** |  |  |  |
|  | *yoaB* | -1,38 | 0,0051 |
|  | *ycnJ* | 1,32 | 0,0066 |
|  | *opuBC* | -1,95 | 0,031 |
| **Sporulation** |  |  |  |
|  | *yjcZ* | -1,95 | 0,064 |
|  | *rapA* | 0,86 | 0,074 |
|  | *gerPF* | -Inf | 0,078 |
| **Ribosome biogenesis** |  |  |  |
|  | *era* | 1,16 | 0,025 |
| **Unknown function** |  |  |  |
|  | *yjdB* | 2,45 | 1,65E-05 |
|  | *oxdC* | 1,66 | 0,00061 |
|  | *yoqM* | 2,54 | 0,00066 |
|  | *ykzT* | -4,05 | 0,00076 |
|  | *ycnI* | 1,59 | 0,0011 |
|  | *yerA* | 1,50 | 0,0038 |
|  | *ytxO* | -Inf | 0,0039 |
|  | *yhcO* | -3,03 | 0,01 |
|  | *yqxI* | 1,29 | 0,011 |
|  | *yozB* | 1,29 | 0,013 |
|  | *yfmQ* | 1,86 | 0,015 |
|  | *ytkA* | 1,61 | 0,019 |
|  | *yvbK* | 2,61 | 0,021 |
|  | *yrzF* | 2,23 | 0,026 |
|  | *ydbL* | 1,14 | 0,038 |
|  | *yvmC* | -2,06 | 0,064 |
|  | *phrA* | 1,23 | 0,067 |
|  | *ydbM* | 1,41 | 0,068 |
|  |  |  |  |
| **Regulon/GO-term** | **gene** | **log2 fold change (*hfq^-^* *vs.* wt)** | **p-value (adjusted)** |
|  |  |  |  |
|  | *ykoJ* | 1,44 | 0,074 |
|  | *ytzD* | 1,20 | 0,093 |
|  | *yebD* | 2,04 | 0,099 |
| **sRNAs** |  |  |  |
|  | sRNA 471 | 0.89 | 0.0035 |
|  | sRNA 2185 | -2.88 | 0.016 |
|  | sRNA 1575 | -0.95 | 0.043 |
|  | sRNA 30 | -1.27 | 0.048 |
|  | sRNA 11 | -1.26 | 0.077 |
| **Type I toxin RNAs** |  |  |  |
|  | *bsrE* | -1.74 | 0.00014 |
|  | *bsrG* | -1.35 | 0.0015 |
|  | *bsrH* | -1.56 | 0.0015 |

**Table S3. *Bacillus subtilis* strains used in this study.**

| **Strain** | **Genotype** | **Source** |
| --- | --- | --- |
| 168 | trpC2 | Laboratory stock |
| GP1067 | trpC2 hfq-Flag spc | This study |
| 168Δhfq | trpC2 Δhfq::*phle* | This study |
| DB104 | *his*, *nprR2*, Δ*aprA3*, *nprE18* | (Kawamura and Doi, 1984) |
| DB104Δhfq::*phle* | *his*, *nprR2*, Δ*aprA3*, *nprE18* Δhfq::*phle* | S. Brantl, Jena, Germany |

**Table S4.** **Oligonucleotides used in this study.**

| Name | Sequence | Destination |
| --- | --- | --- |
| V67_fw | 5′-TT*TCTAGA*ATGAGTAAAGGAGAAGAAC  TTTTCACTGGA-3′ | Construction of plasmid pADΔRBS, cleavage site for *Xba*I (italics) |
| W67_rev | 5′-CTC*AAGCTT*GCATGCCTGCAGGAGAT-3′ | Construction of plasmid pADΔRBS, cleavage site for *Hind*III (italics) |
| F81_fw | 5′-AAG*GAATTC*AGAGCACTGGCGGATCAC  GG-3′ | Construction of *resA-gfp* fusions, cleavage site for *Eco*RI (italics) |
| I88_rev | 5′-AAT*TCTAGA*AGATCCATTCAATTATATC  ATTGCACG-3′ | Construction of *resA-gfp* transcriptional fusion, cleavage site for *Xba*I (italics) |
| J88_fw | 5′- AA*GGAATTC*TATTCGTAAATCTTGTGCT  TGCC-3′ | Construction of *resD-gfp* fusions, cleavage site for *Eco*RI (italics) |
| L88_rev | 5′-AAT*TCTAGA*CTGTTACTTTCATTTTATC  AAATCGTG-3′ | Construction of *resD-gfp* transcriptional fusion, cleavage site for *Xba*I (italics) |
| G81_rev | 5′-AAT*TCTAGA*ACGCCTTTTTTTCTTCATTG  TCC-3′ | Construction of *resA::gfp* translational fusion, cleavage site for *Xba*I (italics) |
| K88_rev | 5′-AAT*TCTAGA*TTCGTTCGTTTGGTCCATG  TTTTCCC-3′ | Construction of *resD::gfp* translational fusion, cleavage site for *Xba*I (italics) |
| M70_fw | 5′-AGGAGAGGGTAAAGAGTGAGAAGCA-3′ | *aprE*, test for the presence of chromosomal DNA |
| N70_rev | 5′-TGAGAGTGAAGAGCCGGCGCTT-3′ | *aprE*, test for the presence of chromosomal DNA |
| Q65_rev | 5′-CCGGATTTGATTCAAAAACTGAT-3′ | Primer extension / *hfq* mRNA |
| B64_rev | 5′-GACTACCATCGGCGCTGAAGAG-3′ | 5S rRNA specific primer |
| O75_rev | 5′-CGGATCTTGATCTGATAGAGG-3′ | Detection of FsrA |
| P75_rev | 5′-GAAGAACAGCGCCCGGGCTAATGG-3′ | Detection of SurA |
| M80_fw | 5′-TTCATTTATGGACCGCTTCTCT-3′ | Detection of sRNA 13 |
| N80_rev | 5′-GGATTCCTCTTTGACAGTGCTT-3′ | Detection of sRNA 13 |
| ML151_rev | 5′-ACA*GTCGAC*TTCGAGTTCAAGCTGGAC  GTTT-3′ | Construction of plasmid pGP1331*hfq*short, cleavage site for *Sal*I (italics) |
| ML152_fw | 5′-ACA*GGATCC*AACCGATTAATATTCAGG  ATCAGTTTTTG-3′ | Construction of plasmid pGP1331*hfq*short, cleavage site for *Bam*HI (italics) |
